# Immersive solutions: South African community service nurses' perspectives on virtual reality potential in hypertension management

**DOI:** 10.3389/fdgth.2025.1430438

**Published:** 2025-03-17

**Authors:** Jayd Brittany Vitorino Clara, Charlene Downing, Patrick Ndayizigamiye, Pieter Herman Myburgh

**Affiliations:** ^1^ Department of Nursing, University of Johannesburg, Johannesburg, South Africa; ^2^ Department of Applied Information Systems, University of Johannesburg, Johannesburg, South Africa; ^3^ Metaverse Research Unit, Institute for Artificial Intelligent Systems, University of Johannesburg, Johannesburg, South Africa

**Keywords:** clinical, hypertension, nursing education, virtual reality, design science research

## Abstract

**Introduction:**

With the rapid development of information technology globally and the scarcity of educators in higher education institutions, educational reforms are crucial to prepare students for an advancing and complex work environment. Virtual reality (VR) makes education widely available as it bridges the gap between students and educators, as educators and students enter an immersive world where educators can guide students.

**Aim:**

The researchers' aim for this study was to explore community service nurses' (CSN's) experiences with a VR prototype when managing a hypertensive patient.

**Method:**

The study comprised nine CSN with varied knowledge, skills, experiences, and who have been allocated to certain disciplines within a public hospital. The study was split into three phases: phase one, focus group and individual interviews were used to gain an understanding of the CSN's current knowledge and experiences regarding the assessment and implementation of nursing interventions used in the management of hypertensive patients. In the second phase, participants were exposed to the VR environment, where they were prompted by the programmed patient avatar to perform several nursing diagnostic procedures and interpret the clinical data provided in order to formulate a nursing diagnosis. During the third phase, the researchers conducted focus groups and individual interviews to acquire and comprehend the participants experiences regarding their interaction with the VR prototype and describe the benefits and drawbacks of the prototype they encountered.

**Results:**

Constructive feedback and recommendations were provided by participants regarding the VR program's interactiveness and the accuracy of diagnostic tests. Participants claimed the experience was enjoyable, and based on the researchers' observations, the VR program stimulated critical thinking as well as clinical reasoning as intended. Their feedback was used to alter the VR prototype before the main study's commencement.

## 1 Introduction

Community service nurses' (CSNs) clinical placement in hospitals is perceived as beneficial and essential. It encourages the development of professional knowledge by amalgamating theoretical knowledge with practical and organisational competencies during the community service year (1). Community service is a crucial 1-year programme for newly qualified nurses (NQNs). After completing their 4-year training as professional nurses and midwives, in accordance with Regulation 174 of 8 March 2013 (2), as amended, as well as the 4-year legacy nursing program R425 as a registered nurse (General, Psychiatric and Community) and midwife (3), NQNs are mandated to complete a community service year before registering as a professional or registered nurse with the South African Nursing Council (SANC), according to Nursing Act No 33 of 2005. This mandatory programme aims to assist newly qualified professional nurses as they transition into their new roles, lessen the effects of transition shock, and potentially bridge the gaps between theory and practice (4–7). While this system has been seen to be effective and is a realistic approach, literature highlights that more needs to be done to better prepare NQN's integrate into their new roles as professionals. NQNs' preparedness has been a historical debate since nursing education transitioned apprenticeship teaching programmes to higher educational institutions regulating and providing nursing courses. These NQNs are expected to “hit the ground running” and perform as proficiently as their senior counterparts (8). Unfortunately, the reality is that these nurses experience anxiety and stress as they transition from a student to professional nurse, attempting to blend into their new roles and apply their learned skills (9, 10). It has also been identified that nursing students generally lack clinical judgement and problem-solving skills, leading to experiential knowledge gaps that hampers a CSN's compliance with SANC's stipulated requirements (11–14). The theory-practice gap represents nursing students and qualified nurses’ inability to integrate their learned scientific theoretical knowledge into experienced clinical practice, as well as the intricacy of interpreting subjective and objective (15), and applying taught nursing principles to render quality nursing care to specific clinical situations (16, 17) and evaluate the outcomes of their nursing interventions (18, 19).

“Clinical reasoning” is defined by Koufidis, Manninen, Nieminen, Wohlin and Silén (20) as the process nurses use to address the health problems a patient experiences at any given moment. It is a cognitive process and is fundamental to the development of clinical judgement. “Clinical judgement”, as defined by Tanner (21), refers to the ability to interpret a patient's needs, delve into their concerns, or identify their health problems. In addition, it includes selecting correct interventions and implementing or adapting a universal approach according to the patient's outcome (21). Therefore, clinical judgement encourages nurses' critical thinking by promoting higher-order thinking skills. It also improves decision-making skills and assists nurses in achieving professional competence and promoting professionalisation, leading to professional independence in nursing (22).

CSNs affirmed that clinical judgement and critical thinking skills are core competencies required to provide quality healthcare to patients. The International Council of Nursing together with Regulation 174 of the Nursing Act No 33 of 2005 (23) acknowledges that nursing competencies consist of the following three domains: knowledge, comprehension, and judgement (24). These skills allow for the identification of observable and subtle abnormalities in patients' health, which a neophyte may not fully recognise (25). As a result of these acquired competencies, experienced nurses have the foresight to diagnose and prioritise patient needs and make prompt decisions as stated by the nurses' scope of practice and midwives (26, 27).

Ultimately, the discord between theoretical knowledge and clinical application, as highlighted by Innes and Calleja (28), can be addressed by using various simulation modalities like virtual reality (VR) to supplement experiential learning (29). VR is an interactive three-dimensional (3D) environment created by computer technology. It combines a multidimensional virtual environment to create a dynamic learning experience by providing a first-person effect and allowing the user to be aware of their spatial presence through several degrees of immersion (30). VR makes education widely available as it bridges the gap between students and educators; educators and students enter an immersive world where students can be guided by educators (31, 32).

VR is extremely beneficial at higher education levels due to its ability to apply numerous learning and teaching theories (33). Learning is an intricate cognitive process involving higher-order thinking, problem-solving, verbal data, concept construction, and information processing. Students ultimately acquire knowledge through mental activities that are formed by internal coding and schemas; therefore, cognitive learning is encouraged (34). VR simulations are also developed using templates with predetermined fundamental elements to learn. Such elements include an overview of the learning purpose, structuring of the scenario, materials and resources, previous learning activities, pre-briefing, assessments, summaries of the scenario, feedback, and debriefing (35). The CSN is able to interact and engage with virtual content, integrating concrete concepts into abstract ideas and facilitating the application of knowledge to real-life scenarios (36). Nursing students gain a better understanding through this process and can integrate newly acquired knowledge based on the concrete experience of interacting with a virtually simulated patient and reflecting on the experience.

In addition to improving nursing skills, VR technologies permit the nurse practitioner to repeatedly practice clinical skills in a controlled patient care environment, allowing the individual to make mistakes safely without potentially devastating outcomes (37, 38). Non-technical skills, such as teamwork, communication, leadership, and the management of stress and fatigue, have also been observed by Peddle, Bearman, McKenna and Nestel (39) to improve due to VR. If global disasters such as the COVID-19 pandemic, combat/war-ridden and infectious areas have taught us anything, it is that higher education faculties have to be resilient and forward thinking. Therefore, VR technologies provide nurses with the opportunity to practice and execute high-risk interventions in a risk-free environment that is self-paced in preparation for the volatile medical field (40–45). This approach is especially beneficial in high-risk clinical settings.

South Africa is experiencing various epidemics of infectious diseases, non-communicable diseases, and trauma (46), and with the substantial incline in chronic diseases, including cardiovascular diseases, the researchers found it fitting to use VR technology to prepare CSNs for the identification and management of hypertensive patients. Cardiovascular disease significantly contributes to the global disease burden in high and low to middle-income countries. According to the World Health Organization (WHO) (47), one in every four men and one in every five women have hypertension. However, the rate of early hypertension detection, treatment and control is less than 20% globally (47). Moreover, hypertension has been proven to be preventable and treatable if diagnosed earlier on. In keeping with the third Sustainable Development Goal of attaining good health and well-being by 2030, the United Nations (UN) hopes to achieve its target of reducing premature deaths caused by non-communicable diseases by one-third through the prevention, treatment and promotion of mental health and well-being (48).

Nurse practitioners' role in treating afflicted patients has evolved from historically merely measuring, monitoring, and recording patients' blood pressure and vital signs, today occupying a more senior position as hypertension specialists (49). Nurse practitioners must consequently continuously develop their theoretical and clinical skills in order to perform diagnostic tasks. This includes, but is not limited to, the detection and treatment of hypertensive patients, the management of prescribed medication, offering patient education, and referrals to further care (50, 51).

## 2 Objectives and research questions

The purpose of this study was to gain an understanding of newly appointed CSNs' experiences using VR to address and evaluate their experiential knowledge gaps. The information was used to formulate recommendations to develop and improve CSNs' clinical reasoning when diagnosing and selecting appropriate nursing interventions to manage hypertension. The following research questions were derived from the research objectives:

Question 1: What are CSNs' experiential knowledge gaps in critical thinking and clinical reasoning related to hypertension?
Question 2: How can immersive VR address these gaps?
Question 3: What are CSNs' experiences of using immersive VR to address their experiential knowledge gaps related to hypertension?

## 3 Materials and methods

The researchers implemented a descriptive phenomenological research method to describe participants' daily experiences of a specific phenomenon to better understand its composition (52). Phenomenological studies explore and create meaning from human experiences by using the descriptions imparted by individuals through comprehensive dialogue (53). In this study, the researchers explored and described CSNs’ experiences using VR to address and evaluate their experiential knowledge gaps.

**Figure 1:**
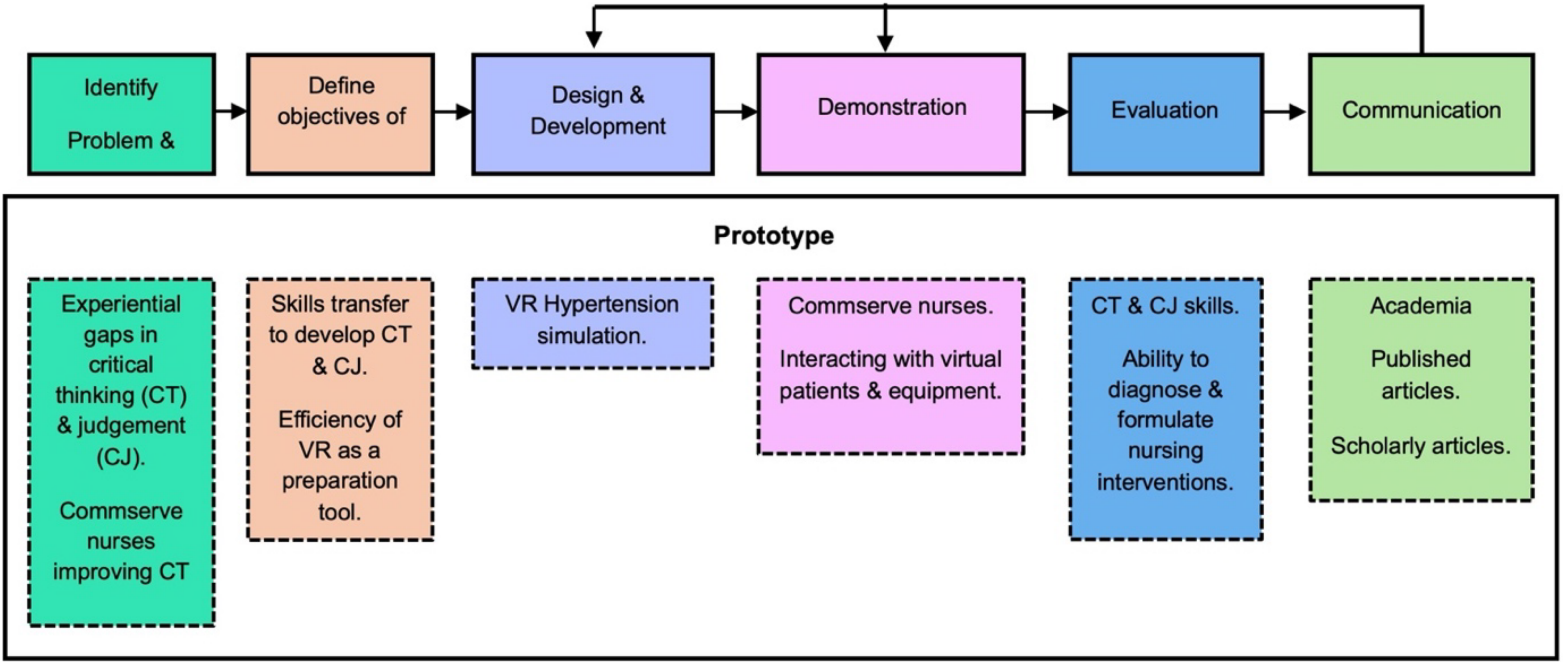
Design science research methodology process.

This study implemented a design science research methodology, as it allowed the researchers to develop an innovative artefact (VR prototype) that was research-specific and designed to creatively solve challenges faced in reality (54). This methodology ensured the objectives of the study were explored in the first five stages, while all findings and recommendations were communicated in the sixth stage through scholarly and published articles, see Figure 1.

### 3.1 Sampling

According to Gray and Grove (55), a population consists of individuals with common characteristics and refers to participants included in the sample. The population of the study comprised nine CSNs who had obtained either a degree or diploma under the legacy programme R425 in general, community, psychiatric nursing, and midwifery, were registered with the SANC, and were actively employed in a public hospital as commserve nurses. Using purposive sampling, participants were selected from a regional healthcare facility in the Ekurhuleni metropolitan municipality in the East Rand of Gauteng, South Africa. The healthcare institution has 230 approved beds but uses 329 and provides specialised healthcare to national and international patriots residing within the Ekurhuleni metropolitan municipality. The following clinical departments are found within this healthcare facility: medicine, surgery, orthopaedics, paediatrics and child health, obstetrics and gynaecology, emergency, anaesthesiology, and radiology. The hospital's disease profile is mainly orthopaedics, surgical and maternal and child health. Therefore, the nine CSNs who met the researchers' inclusion criteria worked in labour, post-natal, theatre, paediatrics, high care, surgical and casualty units. The sample size of 9 participants, although small in scale was deemed sufficient as data saturation was reached as confirmed by both the researchers and an independent coder.

In this study, a moral principle that guided the research included autonomy, which relates to participants' ability to make decisions free from coercion and with full independent competence (56, 57). Participants received an information sheet via WhatsApp and in person explaining the purpose of the study and their expected participation, with the option to accept or decline participation. Another principle adhered to was that of non-maleficence and beneficence, defined as not imposing harm on participants and promoting their well-being by doing good as far as practically possible, as noted by Morrison and Furlong (56) and Pera and van Tonder (57). The researchers ensured that the participants' safety and well-being were prioritised over the purpose of the study by protecting them from activities that may harm them. The principle of privacy was also employed and is defined as the participant's right to non-interference with their personal choices, the confidentiality of their personal information, as well as respect for their personal space and time (57). All personal information was stored in a secure, access-controlled location to prevent the data from being collected and disseminated to third parties. Finally, the principle of justice relates to fairness and the equality of all participants in the study's sample (56, 57). Participants who were interested in partaking in the study were thus selected. Therefore, all interested participants had an equal opportunity to accept or decline participation.

### 3.2 Setting

Ethical clearance for this study was obtained by presenting the study's purpose, objectives, design, and ethical principles to the Faculty of Health Sciences Research Ethics Committee of the University of Johannesburg. The research proposal was reviewed by two independent reviewers. Once necessary alterations were made, the study was approved by the evaluators, resulting in an ethical clearance and renewal certificate being issued. Subsequently, the Higher Degrees Committee was approached for approval, all supporting documentation was submitted, and clearance was provided to commence the study. Permission was also obtained without objection from the Johannesburg Health District Research Ethics Committee, and a permission letter was provided by the specified regional hospital for the researchers to conduct the study at this site. All three phases were conducted at the participants' workplaces according to their availability, and all data were collected by the researchers.

Phase one consisted of one focus group and four individual interviews. The purpose of this phase was to explore and describe participants' experiential knowledge gaps when managing a hypertensive patient. Each session conducted in phase one lasted approximately 30–60 min. The first data collection session was one focus group interview, which consisted of five participants. This focus group was created to accommodate participants' availability and they collectively chose the date and time as they were on the same shift. The next four sessions were individual interviews. The researchers opted to conduct individual interviews due to the availability of participants, as not all participants could attend sessions on the same day and at the same time due to conflicting schedules. The general question posed to participants was to describe CSNs' experiential knowledge gaps. Towards the end of the interview, more specific questions were posed, such as “How would you care for a hypertensive patient?”

Eight of the initial nine participants interacted with the same VR prototype simulation, which mimicked a hospital environment in which they were prompted to collect and analyse subjective and objective data, resulting in the participant having to formulate an accurate nursing diagnosis. The interactive sessions were individually held, allowing the researchers to explain the workings of the VR simulation, provide technical support, and observe each participant's nursing actions while taking notes. Each participant stood with the head-mounted device (HMD) and held handheld toggles in each hand. Once the HMD was fitted comfortably and the participants indicated they were experiencing no visual field blurriness, a simple navigational task (training phase) of moving around in the VR environment, adjusting or manoeuvring their visual fields, and performing basic actions of grasping medical devices and moving them to the patient was performed. This phase lasted 5–10 min. Once the participants indicated that they felt comfortable, the formal VR simulation scenario commenced.

Each interactive session lasted approximately 40–60 min, depending on the participant's ability to formulate a comprehensive nursing diagnosis. During these interactive sessions, the researchers observed the nursing actions each participant took, and, with the aid of field notes and visual recordings, the researchers were able to make notes throughout the sessions. The session was only completed once the participant indicated that they had acquired enough subjective and objective data from their virtual patient and respective diagnostic tests. On completion of this session, each participant received a post-VR interactive questionnaire to record their own nursing diagnosis and rank their nursing interventions in order of importance. Participants were encouraged to take a 5-min break and grab a snack before the commencement of phase three. Seven participants opted to continue with phase three, one requested to commence phase three later, and one did not partake in phase two and was therefore not included in phase three.

One in-person focus group was conducted in phase three, with three participants in attendance. The other four sessions were in-person individual interviews and a telephonic individual interview, as requested by participants according to their availability. Participants who agreed to continue with phase three after completing phase two took a break between sessions and received snacks before the commencement of phase three. The objective of this phase was for participants to reflect on and share their experiences of the VR prototype in terms of managing a hypertensive patient. The focus group and individual interviews lasted approximately 55 min to an hour, and helped the researchers explore CSNs' multifaceted experiences and insights. To start the session, the researchers presented the following topic: “What was your experience of VR in nursing education?” As the interview progressed, more specific questions were posed, like: “What were some of your highlights during the VR interaction?”

### 3.3 Data collection

**Figure 2:**
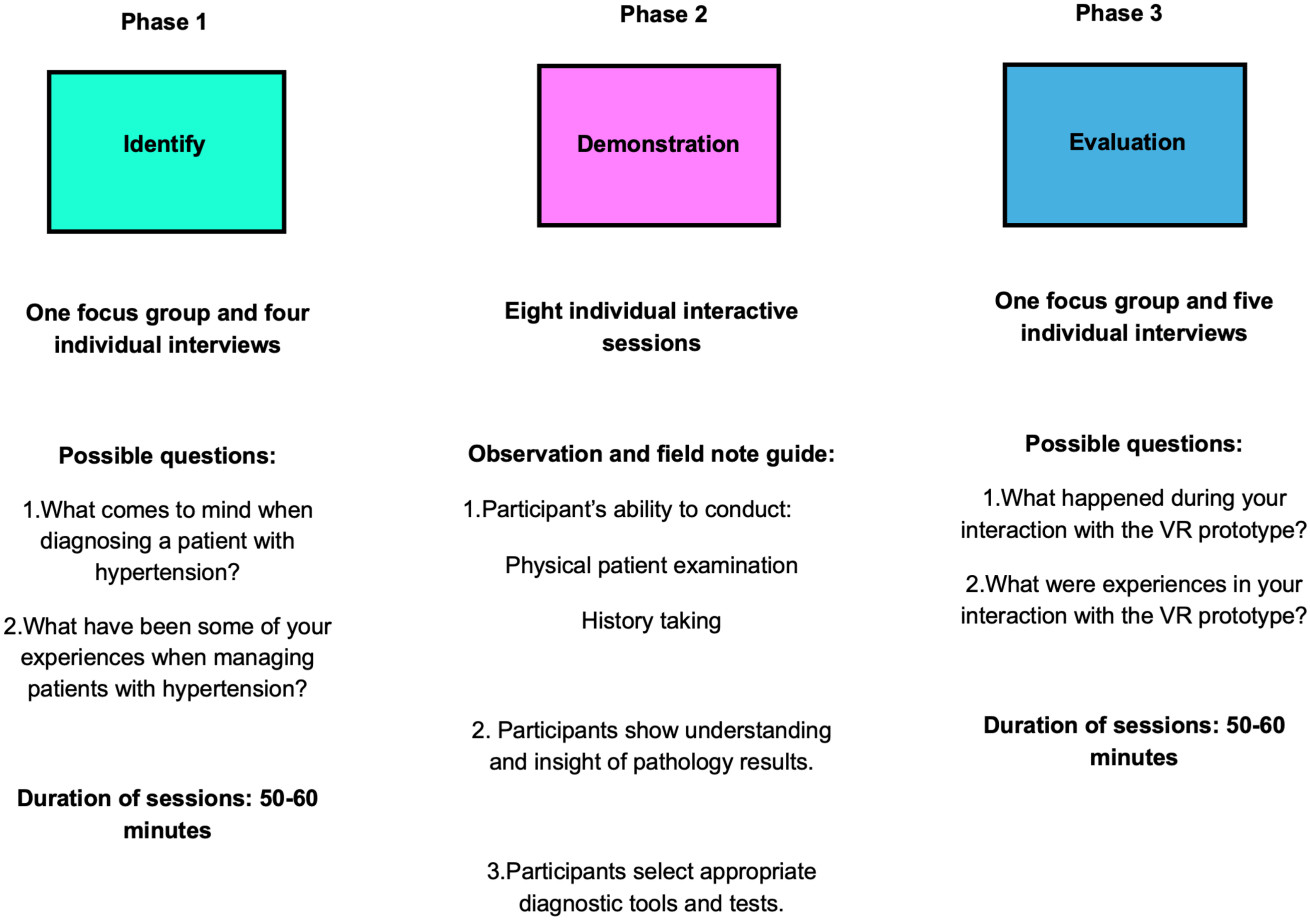
Summary of data collection strategies for the three phases, derived from the design science research methodology process.

Focus groups, semi-structured individual interviews, observations, and field notes were selected as data collection methods. Audio recordings were used in phases one and three, whereas the interactive sessions in phase two were video recorded. Focus groups allowed the participants to discuss the presented topic in a natural environment, helping them to engage and share their ideas with one another, permitting influence from the collective view as opposed to individual viewpoints (53). In addition, focus groups encourage self-disclosure among participants, with the intention of establishing what each member felt and truly thought (58). Semi-structured individual interviews assisted the researchers in gathering thick descriptions of data by asking open-ended questions (59). Although semi-structured interviews follow a pre-established guide or protocol prepared prior to the commencement of interviews, see Figure 2, their main function is to focus on a central topic to provide structure to the interviews (60). Furthermore, this approach provided the researchers with the necessary freedom to pursue topics that became apparent during the interview that they did not initially anticipate (61).

One focus group and four individual semi-structured interviews were conducted in phase one, eight individual participant observations were conducted in phase two, and one focus group and four semi-structured individual interviews were conducted in phase three. Throughout the data collection sessions, the researchers noticed repetitive data and experiences. This allowed the researchers to collect rich and meticulous data and gain an in-depth understanding of CSNs' experiential knowledge gaps when managing hypertension (phase one), and CSNs' experiences of VR bridging these knowledge gaps (phase three).

### 3.4 Data analysis

A thematic data analysis strategy was selected to analyse the data as the researchers could identify recurring meanings throughout a data set, allowing them to interpret the phenomenon (62, 63). Colaizzi's seven-step method was used to interpret the collected data. This data analysis method assisted the researchers in exploring, understanding, and describing CSNs' knowledge and associated gaps. Additionally, the method explored VR's efficiency in addressing these gaps and developing possible recommendations for VR's future use. The process included seven steps: familiarisation with the collected data by transcribing interview recordings and reading through the transcriptions repeatedly to gain insight into the essence of the participants' experiences; identification, extraction and analysis of noteworthy statements relating to the study; formulation of meanings through the identification of central statements to highlight hidden experiences; the identified statements were grouped into theme clusters and further refined into emergent themes; all themes were derived from the data and combined to create a comprehensive description of the data sets; repetitive themes were refined to achieve a succinct description of the data; and once the coding of the data sets was completed by the researchers and the independent coder, themes and categories were validated and confirmed by participants to ensure their experiences were understood and accurately presented (64).

## 4 Results—phase one

Three main themes emerged from phase one, and three main themes emerged from phase three. These findings are illustrated in Tables 1, 2.

**Table 1:** The resulting themes for phase one.

Theme 1	Theme 2	Theme 3
Participants experienced a gap between their theoretical knowledge and clinical practice	Participants experienced a lack of knowledge when having to react instantaneously and appropriately with helpful nursing interventions when treating hypertensive patients	Participants expressed their great need for available and structured mentoring and continued training to integrate theory and practice
1.1 The reality of what participants were expected to know and practice stripped their confidence. 1.2 Participants experienced no mental transition from being a student to suddenly being a professional nurse. 1.3 Participants were ineffectual in performing certain procedures from a lack of practicing these skills. 1.4 Participants were frequently left to their own devices to gain knowledge	2.1 Nursing interventions to manage patients with hypertension. 2.2 Empowering and disempowering factors complicated patient management	3.1 Participants’ needs in terms of continuous training. 3.2 Participants’ needs in terms of the hospital staff

**Table 2:** The resulting themes for phase three.

Theme 1	Theme 2	Theme 3
Participants experienced the VR prototype as very beneficial and exciting to bridge the gap between theory and practice	Participants highlighted the benefit of VR in assessments and curricula	Participants offered recommendations for using the VR prototype to improve the learning process
1.1 The benefits of the VR prototype 1.2 VR contributed to the participants’ development of critical thinking	2.1 Integration of VR as a tool for learning 2.2 VR as a suitable replacement for assessments	3.1 Recommendations to improve the learning process by using the VR prototype

### 4.1 Theme 1: participants experienced a gap between their theoretical knowledge and clinical practice

#### 4.1.1 Subtheme 1.1: the reality of what participants were expected to know and practice stripped their confidence

The participants shared that they felt incompetent and inadequately prepared for the tasks and expectations placed on them without proper knowledge and skill integration. In addition, the participants doubted their abilities and were confused by the differences between what they had learned in theory and what was actually being practised in the clinical setting: “*Especially because the protocol that we use here when treating a patient is different from what we learnt, for example, if you load a patient with Mag Sulph of, what we were taught is that you give it intramuscularly and five grams in each buttock with Lignocaine. Here we don't do that, you give it IV. So, the management is different*” (5 months/Degree/Labour).

Furthermore, certain speciality wards like high care, theatre and casualty departments were new to the CSNs, and they were anxious about suddenly being practitioners and expected to perform as such. This situation robbed many of the participants of the confidence to manage patients’ conditions (including hypertension) efficiently and effectively. It became apparent throughout the interviews that the participants feared the consequences of taking on responsibilities that they knew could lead to a patient's demise: “*But when it comes to specialty wards, for instance, theatre their specialty the high care and casualty, that's where I feel that we have an experiential knowledge gap there because it's like it's a new world together*” (1 year/Diploma/Casualty).

#### 4.1.2 Subtheme 1.2: participants experienced no mental transition from being a student to suddenly being a professional nurse

Participants were aware that although they had the theoretical foundation to manage patients, they were unable to integrate their knowledge into practice: “*So, one of the things that makes it hard for us to practice safely is that, that we don't have the transition for you to move from being a student because once you come to be a commserve. Literally, you still a student, even our mentality isn't advanced or transitioned to being professionals, so we still need to be taken through*” (1 year/Diploma/Casualty). The participants also recognised that they were not sufficiently prepared for what was expected of them as professional nurses: “*So now in the wards I had a little bit of a culture shock because learning about, for example, uh pre-eclampsia in theory is very different to when you actually have a patient in front of you who has a Bp of 220 over like 120 and she starts to fit. It's, it's very different like actually having that real-life person in front of you*” (5 months/Degree/Labour). Moreover, despite participants feeling they lacked the necessary practical skills, they were considered full-workforce employees.

#### 4.1.3 Subtheme 1.3: participants were ineffectual in performing certain procedures from a lack of practising these skills

The participants felt they did not have adequate experience to perform certain procedures, since they were not always allowed to perform or practice them as students: “*So, in xxxx students were not allowed to insert drips and even RNs were not allowed to insert drips. So, it was a little bit hard for us because we couldn't master that skill as, as while we were still students. So, you had to start learning to insert a drip when you are now a commserve in the hospital*” (1 year Diploma/Orthopaedics)*.* Several participants expressed that the theoretical component was emphasised, and they were not always clear on integrating their theoretical knowledge into clinical practice: “*Not confident, especially with ECGs especially because, uhm, number one, it's technical. So, we're not that taught in technical things in college we were just told how to read an ECG but obviously this different manufacturers and you have to know how to operate the ECG machine and the readings as well. Well, I don't quite, I don't remember how to PQRS waves, how do you interpret them and what it means and how does it affect the rhythm, the rhythms and all of that. So, I think for me that that type of skill would be a challenge when I'd be in the situation where there's a hypertensive patient and I don't know, you know uhm how to help and need to nurse that patient*” (1 year/Diploma/Post-natal). Participants alluded that their inability to perform various procedures was attributed to their lack of exposure to certain wards and units, and as CSNs, they were often allocated to a unit in which they had worked 3 years before, which was no longer familiar to them. As a result, they felt their skills, such as operating a ventilator, were unrefined for speciality areas, which led to their significant distress in performing as their senior counterparts.

#### 4.1.4 Subtheme 1.4: participants were frequently left to their own devices to gain knowledge

Some participants shared they would ask for support or assistance from senior professional nurses, and although a few provided the needed support and insight, most senior nurses belittled or ridiculed them in front of patients and their colleagues: “*I get asked certain questions about certain conditions, and I respond in a manner that, you know, using my existing knowledge and then I'm crushed on that. They tell me no go and look it up or we don't care. We don't wanna hear what you learnt at UJ*” (5 months/Degree/High Care). This left the participants feeling incompetent, and they realised they had to take ownership of their learning with the intent to self-learn and self-develop, in turn building their self-confidence. As a consequence of their self-doubt, the participants were motivated to seek guidance and support from alternative platforms like the internet (mainly YouTube), refer back to literature, consult their units' doctors, and seek out unit-specific hospital protocols: “*You also have to like go an extra mile, put more effort, read, watch YouTube videos and then yeah, that's it*” (1 year/Diploma/Post-natal). Participants also stated they reached out to previous CSN groups or colleagues when they experienced incongruencies between theory and practice.

### 4.2 Theme 2: participants experienced a lack of knowledge when having to react instantaneously and appropriately with helpful nursing interventions when treating hypertensive patients

#### 4.2.1 Subtheme 2.1: nursing interventions to manage patients with hypertension

When participants were asked to describe how they would manage a hypertensive patient, a number of them mentioned ensuring the patient was comfortable, nursed in a semi-fowler's position to encourage lung expansion, and within cot sides to prevent the patient from falling. They reported ensuring that the environment was therapeutic to reduce stress for both the patient and themselves so they could anticipate and reflect on the severity of hypertension labouring and post-natal mothers experienced. Other participants said they would start by checking the patient's vitals and saturation levels and inserting a peripheral line and a urinary catheter. Others mentioned the need to gather previous medical history, such as diet, home medication, and the duration of the hypertensive state. Some participants mentioned they would check for pedal oedema and ask if the patient was experiencing any headaches or blurred vision: “*I've already seen from the vital signs that the BP's are elevated. You know that's where you get to ask questions, if the patient sees blurred vision, you are checking the oedema, is no oedema, the headache and stuff then you that's where you need to manage the, the BP's now to try to lower it down*” (1 year/Diploma/Casualty).

The researchers had to probe participants on which other diagnostic procedures they would perform. They then mentioned the doctor would order blood tests, and after further probing by the researchers, some participants considered the need for an ECG but did not feel skilled enough to manage the technical intervention: “*Yeah, maybe ECG, but usually they, they, they prescribe ECG, right. Or we can just do it. Oh. OK, it's in our scope. OK. Yeah. Yeah, do the ECG as well*” (1 year/Diploma/Orthopaedics). Participants did not know how to interpret the PQRST waves in an ECG. A majority of the participants would start administering pharmaceutical drugs and refer to the doctor. Pharmaceutical interventions included the administration of Adalat/Nifedipine or Amloc and magnesium sulphate to labouring or post-natal mothers: “*Usually, what doctors prescribe they start the patient on Amloc 5 milligrams. So, you give the Amloc 5 milligrams per os, and then you'll repeat, I'm not sure that 6 or 8 h*” (1 year/Diploma/Post-natal). The need to provide patients with the necessary health education was also emphasised, mainly concentrating on the need to exercise, adopt healthier diets, reduce or stop tobacco and alcohol use, and the importance of taking anti-hypertensive medications as prescribed: “*Obviously we emphasize diet and managing off stress because obviously I think that's the other part as well the most important, because I think with hypertension, it's not only like uhm only like hereditary and or dietary related, but also with, you know, people with, with this sickness, they obviously have the stressors, illness behavior and then they fail to manage that. So as far as I know, well, yeah, it was that because we gave them like give them like health education on how to manage themselves*” (1 year/Diploma/Post-natal).

#### 4.2.2 Subtheme 2.2: empowering and disempowering factors complicated patient management

Participants reported that their self-doubt was reinforced by managers who inappropriately reprimanded them; this generally entailed being screamed at or reprimanded in front of their patients and colleagues. This behaviour often resulted in patients refusing to be treated by the participant, as they did not trust their abilities: “*She will reprimand you, shout at you in front of patients, like, why is this not done? You're not coping If you don't, you will not survive in my unit. This is the kind of stuff that you get told. If you're not happy, you can leave. That's what I've heard like from other staff members they were told that. So, I think, there was one time where I literally, I nearly lost it. Like I was like I literally wanted to leave. I was like I wish I could just work anywhere*” (5 months/Degree/High Care). Throughout the interviews, participants lamented the lack of committed staff available to train or assist them, and they were instead belittled, ordered to consult their “books”, or go back to school: “*But if you are not expected to do mistakes, where they will be asking what you guys are learning in school (Participant E: Yoh) were they not teaching you. You were dodging*” (1 year/Diploma/Casualty). In addition, the CSNs verbalised that the staff shortage forced them to work several long shifts in a row, and they were not allocated to a specific shift. Participants were used to “fill in the gaps” when the unit was short-staffed, resulting in them feeling exhausted and ineffective.

In addition, the shortage of beds played a crucial role in the CSNs' distress as patients who were deemed critically ill according to their acuity scores were placed in their units. This further added to the participants' stress levels as they were not skilled to nurse critically ill patients and did not receive support or guidance from their senior counterparts. In contrast, a minority of the participants identified that a select few senior staff members were helpful and accessible to ask questions: “*Seniors have been ah, very helpful with regards with hypertension because they, they take you step to step, especially when you find a sister that is a teacher at heart who is not really strict or I don't know how can I put it, but a sister that likes to help others, they really help us when it comes to conditions that we have never seen or we have long forgotten*” (1 year/Diploma/Post-natal). Another participant stated that the multidisciplinary team (allied staff) were very approachable and often offered assistance as they felt sorry for the CSNs: “*But I was, I'm just grateful because the staff here is very supportive. Shame. They taught me a lot and every time I have questions, they are willing, shame, even if they're teaching me something for the tenth time. Yeah, very willing*” (3 months/Diploma/Paediatrics). Ultimately, other staff members' empowering attitudes significantly decreased participants' stress levels.

### 4.3 Theme 3: participants expressed their great need for available and structured mentoring and continued training to integrate theory and practice

#### 4.3.1 Subtheme 3.1: participants' needs in terms of continuous training

The CSNs expressed that they needed staff to be patient, accommodating and professional in guiding them into their new roles as professional nurses. The participants shared they required support and guidance from their seniors to perform their duties within their scope of practice, and they wanted to be reassured that they would not be placed in a position to practice outside their scope. It was apparent that allocated time to ask questions, seek guidance, or receive demonstrations were of great importance to the participants, and increased their confidence in their skills: “*And now you have to see vents. I haven't even seen a vent in my whole life, so I had to see it here for the first time. So, it was quite challenging. But I was, I'm just grateful because the staff here is very supportive. Shame. They taught me a lot and every time I have questions, they are willing, shame, even if they're teaching me something for the tenth time. Yeah, very willing*” (3 months/Diploma/Paediatrics). Furthermore, the participants echoed the need for work environments that are safe, supportive, and facilitative in order to learn new and practice older skills before performing them on real-life patients: “*So that's the thing just giving us an opportunity to make mistakes, be accommodating in that. Once you make a mistake, don't come and hey me, take me through and say OK, this is how you do it like this, like this, because you know, inexperience, experiential knowledge, and theoretical knowledge, they are never the same. You can know everything from the book, but to practice it, it's a different thing*” (1 year/Diploma/Casualty).

#### 4.3.2 Subtheme 3.2: participants' needs in terms of the hospital staff

According to the participants, theory and practice should be equally important. However, they felt the theory component of their training received more prominence, and they were often uncertain how to integrate their theoretical knowledge into clinical practice. A number of participants identified VR's potential benefits in assisting them to manage and provide quality health care in various scenarios: “*That's why I think refresher courses like you say your virtual reality is, ah when you are in commserve, I think it would help a lot of people because if there's, I saw the videos that you sent in the group and I think one was the gestational was it managing pre-eclampsia, I think it was there so that would be very helpful so that you are you are prepared if you're going to be placed in a maternity section*” (1 year/Diploma/Post-natal). They considered VR advantageous to their learning process and emphasised its ability to provide a safe environment to practice their skills. However, some also recognised the value of real-life exposure and would not want that eliminated.

The desire to further develop their skills was obvious as participants highlighted the need for structured learning programmes, such as in-service training, to supplement the orientation programmes they occasionally received: “*I think commserve is meant for you to, it's orientation and transition. Yeah, like you're still orientating yourself into becoming a professional. So, they should allow us the time to like see something, study it and also like have in-services where they like take us into like into depth the practical side of things*” (1 year/Diploma/Post-natal). It was clear to the researchers that the presence of clinical facilitators was deemed important to the CSNs as they emphasised the need to have an allocated professional dedicated to teaching and demonstrating procedures to assist their transition from a student to a professional nurse.

## 5 Results—phase two

The post-VR simulation interaction questionnaire (Annexure A) was created to determine participants' ability to formulate an accurate and appropriate nursing diagnosis for the scenario presented in the VR environment. The questionnaire consisted of two sections: question one asked participants to write down their nursing diagnosis in relation to the clinical scenario and incorporate the subjective and objective data collected within the VR environment. The two main nursing diagnoses derived from the clinical scenario were:

1. Impaired oxygenation due to heart failure and pulmonary congestion as evidenced by dyspnoea, hypoxia (Po2 69 mmHg on ABG), previous tuberculosis, the presence of infiltrates on the chest x-ray, dysrhythmias, decreased oxygen saturation (<90%), pulmonary congestion, abnormal lung sounds on auscultation and fatigue.
2. Decreased cardiac output due to impaired cardiac muscle contraction and increased exertion in workload as evidenced by elevated blood pressures (173/112 mmHg and MAP 135 mmHg), dysrhythmias, hypoxic (PO2 69 mmHg on ABG), chest pains, the presence of abnormal S3 and S4 heart sounds on auscultation, chest pain, presence of abnormal lung sounds on auscultation, dyspnoea, fatigue, and hyponatraemia of 127 mmol/L on ABG.

Participants identified the patient's priority needs were impaired oxygenation and cardiac failure (Annexure B). The participants could relate the objective and subjective data they gathered to the clinical patient presentation. This is an indication that the VR simulation promoted and stimulated participants' clinical reasoning and encouraged participants to develop and refine their clinical judgement, critical thinking, and decision-making skills. As a result, this could potentially enhance NQNs' ability to manage and treat hypertensive patients and improve patient outcomes.

In question two, each participant was asked to arrange the presented nursing interventions in a sequence of importance in order to manage and treat the virtual patient. On completing the interactive session with the VR prototype, each participant was thus asked to complete the provided table and rank their interventions in order of importance or how they would manage a hypertensive patient after interacting with the VR simulation. The significance of this data was to observe the participants' critical thinking, clinical judgement and reasoning when managing a hypertensive patient. The researchers indicated in the far-right column how they would rank their interventions based on their knowledge and experience.

According to the collected data, a majority of the participants indicated they would start by collecting subjective data and a patient history, followed by conducting a head-to-toe physical examination, collecting vital data. Four of the eight participants identified the need to administer oxygen therapy since saturation levels were below the normal range of 90%. In contrast, three participants opted to re-test the blood pressure, and one called the doctor to report the subjective and objective data. Thereafter, the findings for the subsequent management steps were dispersed among administering anti-hypertensives as prescribed by the doctor, performing other diagnostic testing such as an ECG and urinalysis, arranging blood tests, and taking the patient for chest x-ray. Most participants indicated their final step would be to report on and record all their findings and interventions performed on their patients. The data thus illustrated that the participants indeed exercised their critical thinking skills, acted upon their clinical judgement, and were able to rationalise their actions.

## 6 Results—phase three

### 6.1 Theme 1: participants experienced the VR prototype as very beneficial and exciting to bridge the gap between theory and practice

#### 6.1.2 Subtheme 1.1: the benefits of the VR prototype

The participants said the VR prototype was exciting and enjoyable; it inspired further self-learning. Participants also mentioned that VR was a beneficial tool to bridge the theory-practice gaps identified in phase one. It encouraged participants to interrelate their prior knowledge and prompted them to work systematically through subjective and objective data: “*…it made me want to think more logically as if like if this was a real-life situation, what would I do? Uhm like if a patient had to come in and tell me they're having chest pain and they're struggling to breathe. What would be the first thing that you would do? Uhm, and it also helps you like, need to, like, organize my thoughts*” (5 months/Degree/Labour). This skill was essential to identify and prioritise the virtual patient's needs with the intent of formulating a nursing diagnosis and selecting the most appropriate nursing interventions. In addition, all participants shared that the virtual setting provided a safe and supportive environment to practice and refine certain skills with the allowance to make mistakes that would not cause direct harm to a real-life patient. Moreover, participants stated they felt relaxed to make decisions and execute their clinical judgement without worrying about legal and ethical implications: “*…at least I know that it's a safe space to make mistakes and correct them there and there*” (1 year/Diploma/Post-natal).

The VR simulation provided participants with the opportunity to learn and practice new and old skills that they were not necessarily allowed to practice in their clinical settings as students: “*…it was actually really nice to have sort of like a refresher. I especially enjoyed the ECG, uhm because we didn't get much chance to practice that in the hospital as a student*” (5 months/Degree/Labour). Participants emphasised that they particularly enjoyed that the virtual environment provided a private and conducive milieu, which allowed them to feel in control and focus on the task at hand. In addition, the participants expressed that the VR device and program were not complicated to operate, and instructions were clear and easy to work with. They particularly enjoyed and highlighted the benefit of being able to practice ECG lead placements, as well as interpreting x-rays and blood values.

#### 6.1.3 Subtheme 1.2: VR contributed to the participants' development of critical thinking

Participants shared that the VR prototype contributed to their critical thinking and clinical judgement skills by encouraging them to integrate and apply their prior knowledge and skills with the intention of assessing the subjective and objective data in a systematic approach: “*I think the bigger point is that it allows you to systematise your intervention and to prioritise what to do because when you are doing a thing then and there, you actually know and you can actually prioritise and plan, or intervene, immediately. For instance, there was a system of the things you do*” (1 year/Diploma/Casualty). Participants were prompted to identify and prioritise patients' needs, enabling them to formulate nursing diagnoses and prepare nursing care plans by selecting interventions that are specific, measurable, achievable, realistic and time-bound: “*I could actually prioritize, Okay, this, this is what the patient's problems are and from that it's quite easy to literally just draw up a nursing care plan with the problems that are all at hand*” (1 year/Diploma/Post-natal). Similarly, the VR program provided a safe environment for participants to make informed decisions regarding patient care without the added stress of harming the patient: “*It felt like a different world. It's like you are in your own space, you have your own patient. You could experiment and do whatever you want. So, it's a safe space, I would say. Ja. It's a good space, you get to learn and do things at your own pace*” (5 months/Diploma/High Care).

### 6.2 Theme 2: participants highlighted the benefits of VR in assessments and curricula

#### 6.2.1 Subtheme 2.1: integration of VR as a tool for learning

The participants shared their excitement about VR's potential and future use in nursing education. They perceived VR simulation as a valuable and suitable learning tool that should be integrated into nursing practice, as they felt it bridged the gap between theory and practice: “*So, I can definitely recommend a VR to be used in uhm educational institution, especially from health and nursing, it is just going to be, you know, one of the greatest uhm methods to improve the quality of health and of education as well*” (1 year/Diploma/Post-natal). A number of participants expressed that the VR program strengthened their confidence levels as they could immerse themselves in the presented scenario: “*I think even if you just had to use this just to teach students how to place an ECG, I think it would be very useful, also with the, the lung sounds, listening to the lung sounds and the heart sounds to actually be able to hear it was, was really great*” (5 months/Degree/Labour). The participants had an opportunity to interact with various diagnostic instruments and patient information until they were satisfied and confident in formulating a nursing diagnosis. Participants mentioned that they felt the VR simulation program was user-friendly and nurses with varying technological skills would be able to operate the program: “*Ja, and I think this setting, it's more comfortable for old people because they don't know how to use computers, they are not like much acquainted like the younger generation, but they know how to identify a few things*” (1 year/Diploma/Casualty). Consequently, the participants were all in agreement that all categories and age groups of nurses should have an opportunity to develop their clinical skills through virtual simulation.

#### 6.2.2 Subtheme 2.2: VR as a suitable replacement for assessments

During the interviews, participants said they perceived the VR program as extremely beneficial, especially in terms of its possible use as an assessment tool. They shared their excitement that assessment periods or objective structured clinical examinations (OSCEs) would be less stressful, particularly if it meant the preceptor or lecturer did not have to be present in the room with the participant. They attributed their poor performance in these simulation-based experiences to stress and often forgot what they had learned and practised in preparation for the formative or summative assessments: “*…it would really assist you. I think if all assessments were done like that, we will find better results, starting to improve. Because most of them, they are not … it's almost like they are not improving, they are not doing well. They were just intimidated inside, and it was just kicking on and when you come out, you know everything that you didn't do. You just forgot because the lecturer was there*” (1 year/Diploma/Casualty).

Contrary to most of the participants' opinions, one participant expressed the need to still have face-to-face interactions with real-life patients to ensure she is able to perform the necessary procedures confidently and accurately: “*I have a comment on that you asked about the assessment. I think they are good for practice, ja. Training and practice. But at the end final exam, we need to see patients*’ *guys, because yes, it's easy and it's convenient, but when you have to do it, can you actually do it without the virtual reality*” (1 year/Diploma/Post-natal). However, the participant agreed that the VR simulation would be an excellent tool to practice certain skills after interacting with a living patient.

### 6.3 Theme 3: participants offered recommendations for using the VR prototype to improve the learning process

#### 6.3.1 Subtheme 3.1: recommendations to improve the learning process by using the VR prototype

The participants suggested that the VR program should have different difficulty levels. They shared that the entry-level should be easy, with its main focus on orientating the user on the workings of the device and allowing the user to gain a general idea of what they are expected to do. Each subsequent level should progressively increase in difficulty and could even incorporate different scenarios other than treating a hypertensive patient. This would encourage individuals to use and integrate their prior knowledge and stimulate higher-order thinking: “*I think you can up the difficulty level. That was an entry-level for the first year. Ja. So, with … if you are intending to use, so that should be like this, yes. Second year, you get more conditions*” (1 year/Diploma/Post-natal). Similarly, participants requested for some information to be hidden as pop-up icons, as they felt the experience provided them with all the necessary information, and they were almost guided or prompted to use tools in a specific sequence: “*Okay, for me, you know what I was talking about that everything is just there, it's like guiding me what to do. So, somehow it kind of kills your thinking it. So, my suggestion would be that maybe if you can hide some other things, just you come on the screen and you find an option and you will see what to do or you have to see them on the table as they were. Because now like that, it kind of programmes you to say exactly, come here, come here*” (1 year/Diploma/Casualty).

Conversely, several participants expressed that they valued the way the program was set up as it prompted them to work systematically, especially because it was an unfamiliar and newer technology than they were used to operating. The researchers also noted that many of the participants emphasised the need for a more interactive program as they would have appreciated being able to engage in a dialogue with their virtual patient: “*I think as much as the patient was able to give us a history, it would have been nice like to be able to interact like one-on-one with the patient and talk to the patient and ask certain questions*” (1 year/Diploma/Post-natal). Similarly a participant shared,“ *I think that would be very helpful because it would make it also seem more real and you would be able to like actually interact with uhm the patient instead of her just giving you the, the speech in the beginning, it would like it, I think it would just improve the experience if you could talk to her*”(5 months/Degree Maternity).

Another participant expressed the need for a more in-depth introduction to the VR prototype, specifically explaining and illustrating how to use the handheld toggles and allowing more time to acclimate to the virtual environment. One participant suggested a bigger space be used while engaging with the virtual environment to prevent participants from knocking into objects or furniture: “*…also just takes some getting used to, like using the toggle to move things backwards and forwards instead of just being able to walk towards it but maybe if you were in a bigger area that wouldn't be a problem*” (5 months/Degree/Labour). In addition, participants felt that the ease of the VR simulation needed to be improved as they struggled to place some diagnostic instruments in the correct places to generate a response or physiological reading, more specifically the temperate gun: “*Just to improve on the temperature thing starting to, you know, And it should not even guide you on how to put a thing in. You should just use your own feeling inside, what you are going to do, what. Ja and then it can just show you if you have done it wrong. Poef! Maybe there's a sound that it's wrong and to redo, ja. That's what I think should be done.* (1year/diploma/casualty).

## 7 Discussion

The purpose of this study was to explore and describe the phenomenon of CSNs' experiential knowledge gaps and VR's ability to bridge these gaps in managing hypertension. The study acknowledged that CSNs experienced various challenges, from feeling unprepared for their new roles and responsibilities as professionals to experiencing inconsistencies in performing certain clinical skills. Moreover, they feared they might not have the crucial knowledge and skills to render quality nursing care to their patients, experienced a lack of confidence in their skills, and felt they did not have sufficient support and guidance from senior nurses. Consistent with the data collected within this study, Muruvan et al. (7) stressed that as newly qualified professionals, CSNs are expected to function within a new environment that is unfamiliar and multifaceted. Consequently, CSNs are expected to adjust their mindsets to tackle complex, unacquainted responsibilities. Such responsibilities include leadership and organisational tasks, patient care and teaching roles, among others. In addition, Chancey (65) identified several ineffective soft skills contribute to the theory-practice praxis, including time management, communication, stress management, conflict resolution, critical thinking, prioritising, and clinical skills. Similarly, Mabusela and Ramukumba (66) expressed that CSNs were profoundly reliant, incompetent, and exhibited behaviours that were unethical and harmful to patients’ health.

These challenges became apparent during the individual and focus group interviews conducted in phase one, where participants shared their lived experiences. Participants viewed the interview sessions as an opportunity to unpack their perceptions, experiences, and feelings, providing them with a sense of relief at being able to discuss their challenges and ultimately being heard. In addition, participants were concerned about their abilities to provide quality nursing care, specifically in specialised units such as the theatre and intensive care. In support of this observation, Ashipala and Shatimwene (67) and Eklund, Billet and Nilsson (68) further commented that the added pressure to perform meant CSNs are entering a healthcare system that is highly specialised and seeks to provide comprehensive care to communities. It has thus become the norm for hospital managers to place CSNs into highly specialised units such as theatre, intensive care, and oncology due to the shortage of specialised nursing staff (67, 69, 70).

Furthermore, participants shared they would ask for support or assistance from senior professional nurses, and although a few provided the needed support and insight, most senior nurses belittled or ridiculed them in front of patients and their colleagues. Abiodun, Daniels, Pimmer and Chipps (71) confirmed that numerous interpersonal factors, such as bullying, dissension and poor communication are challenges that CSNs encounter within the workplace. Similarly, Abiodun et al. (71) reported that the discouraging attitudes and unsupportive actions portrayed by senior professional nurses toward CSNs threatened the effectiveness and value of this transition period. Conversely, Rosi, Contiguglia, Millama and Rancati (72) highlighted that nurses, specifically senior nurses, do not identify as violent individuals, nor do they believe themselves to be bullies. They rationalise their negative behaviour as a means to test their new colleagues' abilities and skills. They regard this negative attitude as “tough love” and a rite of passage, saying: “we all went through it, and we are here”. Due to their self-doubt, the participants were motivated to seek guidance and support from alternative platforms like the internet (mainly YouTube), refer back to literature, consult their units' doctors, and seek out unit-specific hospital protocols. Participants also stated they reached out to previous CSN groups or colleagues when they experienced incongruencies between theory and practice.

During the study's second phase, the researchers observed that the VR simulation positively affected the participants' critical thinking, clinical reasoning, and judgement skills. Field notes illustrated that the participants were stimulated to use their previous knowledge and experiences and integrate them into the simulated VR scenario to interpret various diagnostic results, prioritise patient needs, and select appropriate nursing interventions. The participants were captivated by the life-like scenario, and most indicated they felt safe to practice and make mistakes without causing harm to the virtual patient. It was evident that past experiences and knowledge influence one's ability to perform optimally, as participants with previous intensive care experience outperformed their counterparts in terms of interpreting arterial blood gases and linking certain test results to disease processes.

During the third phase, CSNs' expectations of VR simulations' use in nursing education and practice were explored. The participants expressed their delight and enjoyment of the virtual experience throughout the individual and focus group interviews. All participants stated they thoroughly enjoyed the interaction and believed it would benefit future student nurses and nurses in practice. They particularly appreciated practising skills in a safe environment without the stress of injuring a patient. Fisher and King (73) shared that clinical simulation, such as VR, provided nursing students with a safe environment to practice their skills, with evidence of knowledge and skills transference to clinical practice. As a result, the authors observed an increase in nursing students' confidence, clinical judgement, theoretical knowledge, and clinical competencies. Furthermore, they expressed that the VR program stimulated them to think systematically, allowing them to draw from their previous knowledge and skills.

Consistent with the collected data, McMullen and McMullen (74) shared the development of critical thinking skills should be regarded as a dynamic process; to develop any new skill, repetition and practice are required to achieve competency. In addition, critical thinking is stimulated by active learning theories and strategies that activate cognitive processes (75). Such learning theories, including experiential learning, propose that immersive technologies can enhance learning performances by presenting students with meaningful and memorable experiences. Multiuser virtual worlds (MUVWs) speak to the experiential paradigm as they provide an environment that fosters engagement and contextual and collaborative learning. MUVWs present opportunities for students to experience collaboration and practice communication, decision-making and problem-solving skills through role-playing. This VR environment supports reflective practice through interactions between the student and facilitator (76). Simulation-based learning is a commonly used and accepted approach in nursing education as it encourages students to integrate theory and practice in order to develop a reflective and questioning attitude (77). By continuously reflecting on their experiences and actively participating in simulated scenarios, students remember their learning material, thereby enhancing their learning (78). These active learning strategies develop and improve students' understanding, stimulate inquiry, and foster critical thinking (79).

Simulations ultimately provide opportunities for nursing students to demonstrate their clinical judgement and prompt them to make decisions in a safe environment while observing consequences and evaluating the effectiveness of their actions first-hand (80). As a result, VR has received increased attention in the field of nursing education and has been implemented to teach and develop higher-order thinking skills among nursing students, including critical thinking, clinical judgement, and decision-making (81, 82). The participants also emphasised VR's benefit if the approach is implemented in practical assessments to remove the anxiety and stressors of having invigilators peering over them. Smith, Farra, Ulrich, Hodgson, Nicely and Matcham (83) added that immersive VR offers a flexible learning environment where nursing students learn at their own pace without the additional burden and stress linked to the presence of faculty members. The participants showed significant enthusiasm and excitedly encouraged the researchers to see this study through so future generations of nurses can benefit from its findings.

With this increased interest in VR among nurse educators, nursing education has been transformed. VR has facilitated a transition of learning from traditional in-person classroom sessions, hybrid, or online asynchronous sessions, to learning on the go and in the comfort of one's home, allowing students to harness their knowledge in practice and learn from mistakes (84). The focus of VR is to enhance students' competencies, and its ability to promote self-directed and blended learning is accentuated. As VR continues to be applied and integrated within curricula, its use will become more readily adopted, and it will become a conventional tool in education. There is no doubt that the demand for the application of virtual simulation will indeed expand in higher education institutions, training facilities, and healthcare organisations due to VR simulations becoming more affordable, requiring fewer resources, and they are not location or time-bound (85). Pottle (81) suggests that simulations should not be an occasional, faculty-led, or day-long event. Students should be able to partake in VR scenarios at their convenience, whether it is at the end of their shift or in the comfort of their own homes. This will motivate students and professionals to continuously improve their clinical competencies and promote continuous professional development. Moreover, as mobile computing and cutting-edge software programs continue to develop, technology such as highly immersive VR systems are increasingly becoming more affordable and accessible to higher education institutions, healthcare organisations and students (86).

The study explored and described CSNs’ experiential knowledge gaps and experiences with VR simulations to bridge these gaps. As a result, the research purpose and objectives were met. However, the researchers also identified several limitations in this study. For instance, the study's sample was limited to CSNs employed at one public healthcare facility. Reduced accessibility to participants also reduced the study's scope as it explored and described eight CSNs' experiences; nevertheless, data saturation was reached. The participants were not trained at the same higher education institutions, which proved to be both a limiting factor as well as a positive one. It limited the study because some participants may have been exposed to more theoretical knowledge, while others had more exposure to clinical practice. Even so, the study's objective was to explore the theory-practice gaps all CSNs experienced. A pilot study of the focus group and individual interviews was also not conducted due to time constraints. However, the flexibility of focus groups and semi-structured interviews meant the researchers still obtained thick descriptions and could pursue topics that emerged during the discussion. Due to time constraints, this study observed participants interacting with the VR prototype for 40 min to an hour. In future studies, a longitudinal study should be conducted to observe the same population over an extended period to better understand or measure the true benefits of VR's application in nursing education. However, important lessons can be learned from this study.

## Data Availability

The raw data supporting the conclusions of this article will be made available by the authors, without undue reservation.
