# Supplementary material for: Immersive solutions: South African community service nurses' perspectives on virtual reality potential in hypertension management
Source: Front Digit Health. 2025 Mar 17;7:1430438. doi: 10.3389/fdgth.2025.1430438 (PMC11955627; doi:10.3389/fdgth.2025.1430438)
Supplement: Supplementary file 1 [file Datasheet1.pdf]

Supplementary Material

Supplementary Material A (Post-Interactive virtual reality simulation questionnaire)

Date:

Participants Name:
Participants Code:

Below is the scenario provided in the VR simulation. Please read through it again and answer the following questions that follow from your experience in the VR environment.

Hello sister, my name is Thandi Modise. I am 46 years of age from Marikana. I am not feeling great, my chest is feeling heavy, and I am short of breath. I have also been experiencing fatigue and sometimes my left arm and chest have been hurting for a short while. The clinic said I have high blood and they gave me tablets but sometimes they have tablets, other times they don't. The tablet also makes me cough. I've stopped smoking now for three years since I had TB. My son, he's now 17, smokes in the house. I do not drink except for special occasions. Then I drink one Savannah and I am done. I do visit a Sangoma sometimes. I am not allergic to anything.

1. **What would your nursing diagnosis be?**

2. **Arrange the following nursing interventions as you would perform them. Indicate a number next to each intervention.**

Sequence	Nursing intervention
	Perform an ECG.
	Administer anti-hypertensive medication as prescribed by the doctor.
	Call and report vital data to the doctor.

	Administer 2L of oxygen via nasal cannula.
	Collect vital data: Blood pressure, heart rate, oxygen saturation, temperature, HGT.
	Report and record actions.
	Conduct head to toe physical assessment.
	Collect subjective data and patient history.
	Re-test Blood pressure.
	Perform the necessary diagnostic tests:<br><br>Blood tests: U&E, FBC, Lipid profile & BUN<br><br>CXR<br><br>Urinalysis

Supplementary Material B (Participants responses to the post-interactive virtual reality simulation questionnaire).

Question 1

The two main nursing diagnoses derived by the researcher from the clinical scenario were:

1. Impaired oxygenation due to heart failure and pulmonary congestion as evidenced by dyspnoea, hypoxia (Po2 69mmHg on ABG), previous tuberculosis, the presence of infiltrates on the chest X-ray, dysrhythmias, decreased oxygen saturation (<90%), pulmonary congestion, abnormal lung sounds on auscultation and fatigue.
2. Decreased cardiac output due to impaired cardiac muscle contraction and increased exertion in workload as evidenced by elevated blood pressures (173/112mmHg and MAP 135mmHg), dysrhythmias, hypoxic (PO2 69mmHg on ABG), chest pains, the presence of abnormal S3 and S4 heart sounds on auscultation, chest pain, presence of abnormal lung sounds on auscultation, dyspnoea, fatigue, and hyponatremia of 127mmol/L on ABG.

The following responses were provided by the participants for question one:

Participant A: Impaired circulatory need related to elevated BP as evidence by the patient reporting having a heavy chest.

Participant B: Cardiac attack/failure related to undiagnosed elevated hypertension as evidenced by the patient verbalizing feeling heaviness in the chest and left arm and chest pains.

Poor airway circulation related to medical disease process as evidenced by patient verbalizing shortness of breath (SOB) and saturation (sats) of 86% on room air (RA).

Participant C: Query myocardial infarction (MI) related to patient's high blood pressure as evidenced by chest pains and pains in the left arm as verbalized by the patient.

Participant D: Inadequate cardiac output related to persistently elevated BP as evidenced by enlarged heart on chest X-ray.

Participant E: Participant did not participate.

Participant F: Compromised cardiac output related to enlarged and thickened ventricles and heart, as evidenced by ECG, protein 2+ in urine and blood trace in urine, drop in Hemoglobin (HB), elevated BP, decreased saturation, and patient reporting radiating chest pain.

Participant G: Short of breath related to lung infiltrates as evidenced by patient verbalizing heavy feeling in chest and difficulty breathing.

Increased BP related to cardio vasoconstriction as evidenced by chest X-ray indicating the heart has enlarged.

Participant H: Increased cardiac output related to underlying condition as evidenced by patient having BP: 178/87, MAP 135, HR (heart rate) 83, patient having pain in the left arm and chest, chest feeling heavy and K+ 4.4 mmol/L.

Ineffective tissue perfusion related to hypertrophy as evidenced by patient experiencing fatigue, sats of 87% on RA, shortness of breath, P/F ratio of 196.

Participant I: Elevated blood pressure related to non-compliance as evidenced by a systolic of 184 and a diastolic of 104 and patient verbalizing that sometimes the clinic does not have the tablets.

Question 2:

**Participants**	**A**	**B**	**C**	**D**	**F**	**G**	**H**	**I**	**Researcher**
**Nursing intervention**	**Sequencing of nursing interventions**								
Perform an ECG.	8	5	7	9	9	7	6	8	5
Administer anti-hypertensive medication as prescribed by the doctor.	7	7	9	6	7	6	9	6	7
Call and report vital data to the doctor.	9	6	6	8	5	4	7	5	6
Administer two litres of oxygen via nasal cannula.	4	4	3	5	6	9	4	4	2
Collect vital data: Blood pressure, heart rate, oxygen saturation, temperature, HGT.	2	3	2	3	3	2	3	3	4
Report and record actions.	10	9	10	7	10	10	10	10	10

Conduct a head-to-toe physical assessment.	5	2	5	2	2	3	2	2	3
Collect subjective data and patient history.	1	1	1	1	1	1	1	1	1
Re-test Blood pressure.	3	10	4	4	4	5	5	7	8
Perform the necessary diagnostic tests:<br>Blood tests: U&E, FBC, Lipid profile & BUN<br>CXR<br>Urinalysis	6	8	8	10	8	8	8	9	9

Table 1. Participants' and researchers ranking of nursing interventions.
